# Efficacy of Precocene I from *Desmosstachya bipinnata* as an Effective Bioactive Molecules against the *Spodoptera litura* Fab. and Its Impact on *Eisenia fetida* Savigny

**DOI:** 10.3390/molecules26216384

**Published:** 2021-10-22

**Authors:** Narayanan Shyam Sundar, Sengodan Karthi, Haridoss Sivanesh, Vethamonickam Stanley-Raja, Kanagaraj Muthu-Pandian Chanthini, Ramakrishnan Ramasubramanian, Govindaraju Ramkumar, Athirstam Ponsankar, Kilapavoor Raman Narayanan, Prabhakaran Vasantha-Srinivasan, Jawaher Alkahtani, Mona S. Alwahibi, Wayne Brian Hunter, Sengottayan Senthil-Nathan, Krutmuang Patcharin, Ahmed Abdel-Megeed, Rady Shawer, Aml Ghaith

**Affiliations:** 1 Division of Biopesticides and Environmental Toxicology, Sri Paramakalyani Centre for Excellence in Environmental Sciences, Manonmaniam Sundaranar University, Alwarkurichi 627 412, India; krn.shyamsundar@gmail.com (N.S.S.); karthientomology@gmail.com (S.K.); sivanesh2020@gmail.com (H.S.); stanleyrajamsu@gmail.com (V.S.-R.); clairdelune127@gmail.com (K.M.-P.C.); heyram.8238@gmail.com (R.R.); ayvidram@gmail.com (G.R.); 2 Department of Biotechnology, Sri Paramakalyani College, Alwarkurichi 627 412, India; sreeram.pons@gmail.com; 3 Department of Zoology, Sri Paramakalyani College, Alwarkurichi 627 412, India; kraman.narayanan@gmail.com; 4 Department of Biotechnology, St. Peter’s Institute of Higher Education and Research, Avadi 600 054, India; vasanth.bmg@gmail.com; 5 Department of Botany and Microbiology, College of Science, King Saud University, Riyadh 11451, Saudi Arabia; jsalkahatani@gmail.com (J.A.); wamona015@gmail.com (M.S.A.); 6 USDA-ARS, Agricultural Research Service, U.S. Horticultural Research Laboratory, 2001 South Rock Road, Fort Pierce, FL 34945, USA; wayne.hunter@usda.gov; 7 Department of Entomology and Plant Pathology, Faculty of Agriculture, Chiang Mai University, Chiang Mai 50200, Thailand; patcharink26@gmail.com; 8 Innovative Agriculture Research Center, Faculty of Agriculture, Chiang Mai University, Chiang Mai 50200, Thailand; 9 Department of Plant Protection, Faculty of Agriculture Saba Basha, Alexandria University, Alexandria 21531, Egypt; rady.shawer@alexu.edu.eg; 10 Department of Zoology, Faculty of Science, Derna University, Derna 417230, Libya; moly2gm@gmail.com

**Keywords:** armyworm, botanicals insecticides, phytochemical, toxicity, Noctuidae, histology

## Abstract

The sustainability of agroecosystems are maintained with agro-chemicals. However, after more than 80 years of intensive use, many pests and pathogens have developed resistance to the currently used chemistries. Thus, we explored the isolation and bioactivity of a chemical compound, Precocene I, isolated from the perennial grass, *Desmosstachya bipinnata* (L.) Stapf. Fractions produced from chloroform extractions showed suppressive activity on larvae of *Spodoptera litura* (Lepidoptera: Noctuidae), the Oriental armyworm. Column chromatography analyses identified Precocene I confirmed using FTIR, HPLC and NMR techniques. The bioactivity of the plant-extracted Dp-Precocene I was compared to a commercially produced Precocene I standard. The percentage of mortality observed in insects fed on plant tissue treated with 60 ppm Db-Precocene I was 97, 87 and 81, respectively, for the second, third and fourth instar larvae. The LC_50_ value of third instars was 23.2 ppm. The percentages of survival, pupation, fecundity and egg hatch were altered at sub-lethal concentrations of Db-Precocene I (2, 4, 6 and 8 ppm, sprays on castor leaves). The observed effects were negatively correlated with concentration, with a decrease in effects as concentrations increased. Distinct changes in feeding activity and damage to gut tissues were observed upon histological examination of *S. litura* larvae after the ingestion of Db-Precocene I treatments. Comparative analyses of mortality on a non-target organism, the earthworm, *Eisenia fetida*, at equal concentrations of Precocene I and two chemical pesticides (cypermethrin and monocrotophos) produced mortality only with the chemical pesticide treatments. These results of Db-Precocene I as a highly active bioactive compound support further research to develop production from the grass *D. bipinnata* as an affordable resource for Precocene-I-based insecticides.

## 1. Introduction

Agricultural productivity is drastically reduced due to insect pests, with an estimated annual post-harvest loss of about 10–30% of major agricultural crops [1]. Climatic conditions in a tropical country such as India favor the growth of agriculture pests, which in turn affect the productivity [2]. Growing economic and environmental concerns have increased interest in the use and development of less persistent, botanical-based insect pest management products. The unselective usage of chemical pesticides results in the development of pests with genetic resistance to insecticides [3], while posing potential hazards with toxic residues, environmental health hazards and a negative impact on beneficial insects, such as predators, parasitoids and pollinators [3]. Phytochemicals in plants offer a huge, untapped variety of chemicals that have potential use to defend crops against agricultural pests [4]. Some botanicals have displayed significant targeted activity against different pests [5]. Common effects on insects from crude extracts or semi-purified isolated fractions of phytochemicals report disruption of development, inhibition of growth by altering expression of the growth regulating hormones or digestive enzymes. Phytochemicals may also produce tissue malformations, adult sterility or increased mortality usually in a dose-dependent manner [6]. Plant phytochemical researchers have identified around 10,000 secondary metabolites [7]. Planning programs for establishing an effective botanical pesticide economy suggest several basic principles: (1) for sustained production, the chosen plants should grow year round, (2) plants must provide significant biomass to yield suitable concentrations of the extracted active compounds and (3) the proper extraction methods for producing a final product that is economically feasible and stable in the final product composition [7]. Indicators of plants that contain potential suitable phytochemistry are identified by being unpalatable, bitter or caustic. Common chemicals from plant with these traits include alkaloids, flavanoids, phenols, quinone, terpenes and coumarin-like compounds, all of which have also been documented to exhibit some level of activity against agricultural pests [3,6].

The destructive feeding behavior of phytophagous larvae in the orders Lepidoptera and Coleoptera is well documented to cause excessive damage to agricultural crops [8,9,10]. Continuous developments of insecticides or antifeedants such as silicon for pest management are greatly needed. Some of the most significant advances are coming from studies on the effects from secondary plant metabolites [11].

The lepidopteran, *Spodoptera litura*, commonly called Oriental armyworm, tobacco cutworm, cotton leafworm or taro caterpillar [12], is a member of the devastating “Armyworms” [13]. Polyphagous in nature, these lepidopteran pests are cosmopolitan in distribution [14], with *S. litura* found predominantly in tropical countries [15]. The larvae are avid feeders with over 150 host species [16]. They extensively infest more than 112 species of crop plants in India [17]. While not a seasonal pest, there may be 7 to 8 generations per year [18] and 12 generations in central India [19]. They infest crops, viz., cotton, groundnut, chilly, tobacco, caster and pulses in India, China and Japan and occur in Australia and New Zealand [20]. The larvae damage seedlings of many crop plants, resulting in severe economic loss [21,22]. The global annual economic loss from this pest has been over USD 2 billion of which 80% of the loss occurs in India alone [23]. High loss of yield has been directly related to high density levels of *S. litura* [24] resulting in high levels of defoliation and plant death [25]. Development of insecticide resistance, i.e., an increase of 2700-fold against pyrethroids [26,27,28], has resulted in inadequate control of these lepidopteran pests [5]. New solutions are desperately needed against these pests.

*Desmostachya bipinnata* L. is a perennial grass, in the taxonomic family Poaceae. The phytochemical constituents of this plant [29,30] and pharmacological uses are well studied [31]. Plant seed extracts have an effective microbiocide. [32,33], though the role of this plant as an insecticide has not received much study, particularly on the lepidopteran insect pest, *S. litura.* In terrestrial ecosystems, earthworms are important soil fauna that improves the soil matrix and physical properties by decomposing organic materials [34]. Earthworms are used as the index of soil quality; thus, they serve as candidates to study the acute and chronic toxicity of chemical treatments in agricultural settings [35,36]. Toxicological effects of chemicals on various aspects of earthworm such as growth and survival rate, reproductive efficiency, apoptosis nature of cell, membrane stability of lysosome, concentration of metallothionein, activity of enzyme and immune system and DNA damage have been conceded out [37,38,39].

Application of chemical pesticides not only kills the insect pest but may also affect many non-target organisms [40]. Thus, the earthworm, *Eisenia fetida* was chosen as the non-target organism in this study, were easily availability, with established protocols for rearing, and toxicity bioassays [41]. The present research reports the effects of Precocene I treatments on leaves fed to *S. litura* on survival, fecundity, egg hatch, gut histological and development, while also evaluating toxicity on a non-target organism, an earthworm.

## 2. Materials and Methods

### 2.1. Chemicals

The solvents (hexane, chloroform, ethyl acetate, toluene, formic acid and methanol) used in this experiment were bought from Merck (Darmstadt, Germany), and all the solvents used were of HPLC grade. The standard compound Precocene I (≥95% purity), synonym: 7-Methoxy-2,2-dimethyl-3-chromene, Empirical Formula (Hill Notation) C_12_H_14_O_2_ was obtained from Sigma Aldrich, Mumbai, India.

### 2.2. Plant Sample Collection

The plants *Desmostachya bipinnata* were collected at the river banks of Kadana Dam at Sivasailam (latitude, 8° 39′ 57.01″ N; longitude, 77° 35′ 18.23″ E; elevation, 62 m), Tamil Nadu, India. The collected plants were confirmed with regional flora (Gamble, 1934), and then, voucher specimens were submitted to the SPKCES, Manonmaniam Sundaranar University, Tirunelveli, India.

### 2.3. Crude Extract Preparation

The leaves of *Desmostachya bipinnata* were shade-dried at room temperature. The dried leaves were powdered and sequentially extracted following the method of Ponsankar et al. [40] with slight modifications. They were extracted serially by using methanol, chloroform and petroleum ether. The solvents in desiccators were removed, and the residues were stored under 4 °C for further analysis.

### 2.4. Isolation of Bioactive Compound

The crude chloroform extract (50 g) was dissolved in 100 mL of 90% ethanol, and aqueous extracts were made from this by adding 200 mL of distilled water. The fractionation of aqueous extract was performed with column chromatogram with silica gel (60–120 pore size). The elution of column was performed with step gradient mixture of ethyl acetate and ethanol. The collected eluates were fraction I (ethyl acetate), fraction II (ethyl acetate/ethanol, 11:1, *v*/*v*), fraction III (ethyl acetate/ethanol, 10:2, *v*/*v*), fraction IV (ethyl acetate/ethanol, 9:3, *v*/*v*), fraction V (ethyl acetate/ethanol, 8:4, *v*/*v*), fraction VI (ethyl acetate/ethanol, 7:5, *v*/*v*), fraction VII (ethyl acetate/ethanol, 6:6, *v*/*v*), fraction VIII (ethyl acetate/ethanol, 5:7, *v*/*v*), fraction IX (ethyl acetate/ethanol, 4:8, *v*/*v*), fraction X (ethyl acetate/ethanol, 3:9, *v*/*v*), fraction XI (ethyl acetate/ethanol, 2:10, *v*/*v*) and fraction XII (10% ethanol). Further, twelve fractions were evaporated at room temperature for complete dryness.

The eluted fractions were applied on TLC (60 F254 Merck, Germany) containing formic acid, water, ethyl acetate and toluene (ratio (*v*/*v*) of 3:3:25:50), and eluted samples were compared with the standard Precocene I. The TLC plates were squirted with stannous chloride in dilute HCl of 100 g/L and for 1 min heated at 100 °C. The spots obtained were visualized under UV light at 365 nm and compared with the standard Precocene 1 for the identification of the structure. Fraction VII showed a similar Rf value with that of the standard, Precocene I, and the partly purified fraction VII was recrystallized using methanol to yield the extracted pure compound of *D. bipinnata,* Db- Precocene I. The eluted VII fraction was analyzed using HPLC studies followed the method of Ponsankar et al. [42]. The characterization of the compound structure was carried out by FT-IR and NMR spectral analyses.

### 2.5. Fourier Transform Infrared Spectroscopy Analysis

The Fourier transform infrared spectroscopy (FT-IR) spectra of fraction VII were observed on Perkin Elmer Spectrum one equipped with an ATR-FTIR unit. A small quantity of the fraction VII was kept in the ATR head, and the recording of spectra carried out at wavelengths from 450 to 4000 cm^−1^. The spectra resolution was found at 4 cm^−1^, and sixteen scans were ensued and averaged. Separate software used for analyzing the data observed in the spectrum (Perkin Elmer, Vadodara, India).

### 2.6. NMR Analysis

Fraction VII was subjected to characterization through NMR spectral analysis [42]. The NMR spectra ^1^H and ^13^C of the isolated compounds were recorded at 300 mHz (^1^H NMR) and 75 mHz (^13^C NMR) employing Bruker (Advance) NMR instrument in CDCl_3_ solvent with 5–10 drops of dimethyl sulfoxide (1 drop ~200 mL). The chemical shifts were referenced to tetra methyl silane and expressed in parts per million.

### 2.7. Insect Culture

The collected *S. litura* pupae were cultured in the laboratory, Biopesticide and Environmental Toxicology. The emerged adults were transferred to the sterile elastic container containing leaves harvested from castor plant for oviposition and were nursed with 8% of honey solution dipped in the cotton wool and placed inside the small cap of glass. The laid eggs were retained for hatching in sanitized plastic containers and placed in the ecologically controlled chamber. The required temperature and relative humidity (RH) were maintained in the chamber as 25–27 °C, at 60–70%, respectively. The day/night time was fixed to 13:11 h [27,41].

### 2.8. Mortality Bioassay

Mortality bioassay was carried out on II, III and IV instars larvae of *S. litura* using discriminate dosages (5, 10, 15 and 50 ppm) of fraction VII (suspended in 0.1% chloroform). Leaves of castor were provided with set concentrations of fraction VII and air dry for five minutes. For control, chloroform of 0.1% was sprayed on the castor leaves. The 4-hour starved II, III and IV instar larvae were fed with the fraction-VII-treated leaves (5, 10, 15 and 50 ppm), and the experiments were carried out in a sterilized room (27 ± 2 °C) with a relative humidity (RH) of 80%. Ten larvae were taken for each concentration. The procedure was passed out with five replicates, and one control group was maintained.

(1) $$Corrected\;percentage\;of\;mortality=(1-\frac{n\;in\;T\;after\;treatment}{n\;in\;C\;after\;treatment})\times 100$$

(2) $$Precentage\;Mortality\;\left(\%\right)=\frac{Number\;of\;larvae\;dead}{total\;number\;of\;larvae\;introduced}\times 100$$

Mortality rate was recorded at an interval 24 h and compared with control. The mortality rate of percentage was studied using Equation (1), and corrected percentage mortality was carried out by using Abbott’s formula [42], Equation (2), where *T* is the number of larvae in treated groups; C is the number of larvae in control groups.

The corrected data of mortality were exposed to Probit analysis [43] to determine the lethal concentration (LC_50_ and LC_90_). Developmental aspects were studied using the sub-lethal dosage.

### 2.9. Development Studies

Developmental aspects were studied following the procedure of Senthil-Nathan and Kalaivani and Selin-Rani et al. [21,44]. The second instar emerged larvae were used for biological studies. The starved larvae were placed with castor leaves treated with 6 ppm of fraction VII. Control were provided with 0.1% chloroform-treated leaves and let dry. Every 24 h, leaves that were not eaten were detached and fresh castor leaves were added. A minimum of ten larvae was maintained for each concentration with five replicates. The treated and control groups were kept in 14L:10D conditions at 28 ± 2 °C and 80% RH. Every 24 h, removal of dead larvae and recording of live individuals were carried out. Fecundity was assessed by noting the emerged moths from the treated larvae. They were further raised with normal adults of the differing sex from the in vitro cultures to observe the progeny number generated, and then, pairs were limited in cover containers with interned castor leaves for oviposition.

### 2.10. Antifeedant Activity

Activity of antifeedant of fraction VII was tested by using the leaf disc choice method [45]. Fraction VII with concentrations of 5, 10, 25 and 50 ppm was used to evaluate their potentiality in view to find the cessation ability on feeding activity against V instar of *S. litura*. Leaf discs of 3.5 cm diameter from castor plant were treated on both sides with 20 μL of fraction VII (5, 10, 25 and 50 ppm) and, then, air-dried at room temperature. Leaves of control were treated with 0.1% chloroform. Disc leaf were reserved in petri dish (15 cm) lined with moistened filter paper. Four-hour-starved V instar were introduced in a petri dish containing control and untreated leaf disc. Five replications were conceded out in each concentration. The treated and control sets were placed in 14L:10D (28 ± 2 °C and 80% RH). The Feeding Deterrence Index (*FDI*) was calculated by employing Formula 3.

(3) $$FDI=\frac{C-T}{C+T}\times 100$$

where *C* and *T* are the control leaf and treated leaf weights consumed by the *S. litura* [46].

### 2.11. Histology Study

The control and Precocene-I-treated larvae were longitudinally cut through the cuticle and fixed in 4% formaldehyde for 2 h at 4 °C following the method of Raymond et al. [47] and with meagre modification as specified by Pradeepa et al. [48]. The tissue was dehydrated in ascending grades of alcohol, viz., 50, 60, 70, 80, 90 and 100% for every two hours and put in xylene for 6 h. Then, they were placed in a warm oven and embedded in paraffin to prepare blocks. Sections of about 8mm inches were taken and dewaxed by dipping 5 min in 100% xylene. The rehydrated tissues in descendant grades of alcohol, viz., 100, 90, 80, 70, 60 and 50% and, then, with distilled water. They were stained with Ehrlich’s hematoxylin and again dehydrated with descending grades of alcohol as mentioned above and counterstained with eosin. The slides were washed once with 100% alcohol and dipped twice in xylene. Samples were mounted with DPX [49]. Observation on the midgut of control and treated larvae was made by a Nikon microscope (Japan), and the microscope was connected to a computer to the photomicrograph.

### 2.12. Earthworm Culture

Adult earthworms, *E. fetida*, were reared in the BET laboratory, M.S. University, India, without exposing them to pesticides. A soil mixture and manure of cattle contained wooden box *E. fetida* were introduced and cultured. Distilled water was sprayed in the wooden box to maintain the temperature (28.9 ± 0.36 °C) and moisture (30%). Adult earthworms (350–430 mg) with clitellum were used for experiments [24].

### 2.13. Non-Target Acute Toxicity Assay

The artificial soil was taken for testing [39,44]. The content of the soil was 70% quartz sand, 20% clay of Kaolin and 10% peat sphagnum, and the pH was maintained at 6.0 by adding calcium carbonate. To 250 mg/kg of soil, different pesticide concentrations (basis of dry/weight) such as chlorpyrifos, cypermethrin and Db-Precocene I were added. The selected amount of pesticide was mixed into soil in the form of aqueous solution to obtain the working soil.

### 2.14. Statistical Analysis

The statistical analyses of data from larvicidal assay, antifeedant and biological studies were carried out using an ANOVA variance (percentage of square root transformation). The Tukey’s family significant error rate by Minitab^®^17 statistical software (Minitab, Inc., State College, PA, USA) determined the significance between the treatments. If *p* ≤ 0.05, then the variance between means was significant (Snedecor and Cochran, 1989). The Probit analysis with accountable interval of 95% using the Minitab^®^ 17 program was accompanied to calculate the lethal concentrations necessary to kill 50% (LC_50_) of larvae in 24 h. The Abbott’s (1925) equation was utilized to correct the mortality if it was needed.

## 3. Results

### 3.1. Identification of Db-Precocene I

The bioactive compound Db-Precocene I was isolated from chloroform extract of *D. bipinnata* with the help of chromatographic techniques and was subjected to TLC, FT-IR, HPLC, ^1^H NMR and ^13^C-NMR for compound identification. The TLC profile of Db-Precocene 1 from chloroform extract showed similar retardation factor (*Rf*) value (*Rf* = 0.682) when compared with the standard Precocene I.

### 3.2. Mortality Bioassay

**Figure 1:**
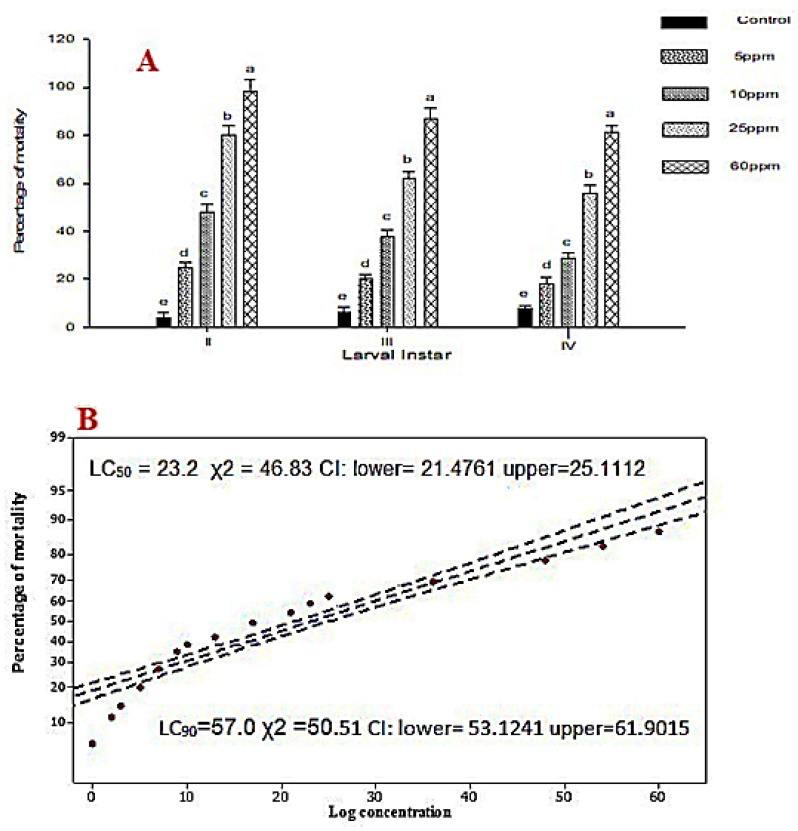
(**A**) Percent larval mortality was found to be pronounced in second, third and fourth instars of *S. litura*. Means (± (SE) standard error) followed by the same letters above bars indicate no significant difference (*p* < 0.05) according to Tukey’s test. (**B**). Median lethal concentrations (LC_50_ and LC_90_) of Db-Precocene I against fourth instar of *S. litura* Probit analyses.

The larval mortality was found to be pronounced in second, third and fourth instars of *S. litura* when they were exposed to the isolated Db-Precocene I (Figure 1A). The II, III and IV instars of *S. litura* showed higher mortality at 60 ppm concentration of Db-Precocene 1 than at 5, 10 and 25 ppm. The mortality rate increased with prominent treated dosage (5 ppm, 10 ppm, 25 ppm, 60 ppm) producing a dose-dependent mortality rate.

The LC_50_ value of Db-Precocene I against the third instar larvae was calculated to be 23.2 ppm (Figure 1B) the Db-Precocene I was tested against the *S. litura* for toxicity; at 60 ppm, the concentration produced a significant mortality of 98% (*F*_4,21_ = 49.73, *p <* 0.001), 87% (*F*_4,21_ = 55.18, *p <* 0.001) and 81% (*F*_4,21_ = 52.21, *p <* 0.001) in the second, third and fourth treated larvae. While it was 80%, 62% and 56%, respectively, at the concentration of 25 ppm. Whereas, in 10 ppm, 48%, 38% and 29% of mortality was recorded in second, third and fourth instars. Even at the concentration of 5 ppm of Db-Precocene I, significant mortality was observed greater than the control (*p* < 0.05).

### 3.3. Development Studies of S. litura

**Figure 2:**
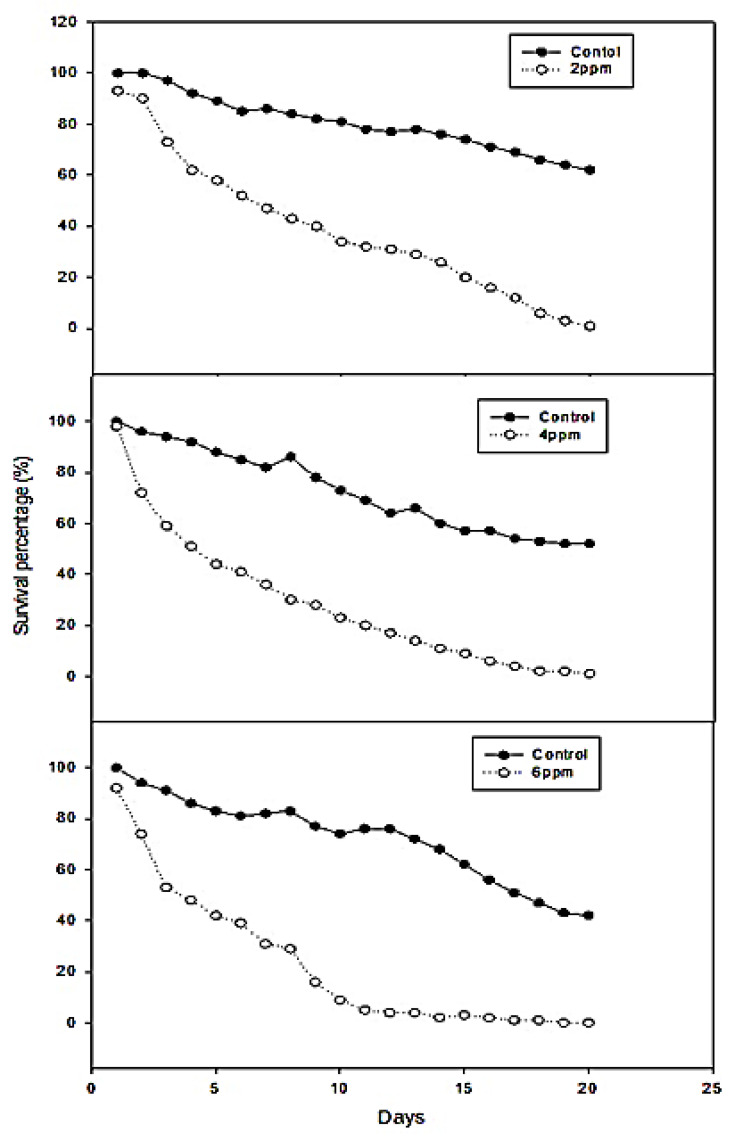
Mean percentage larvae mortality at 20 days after treatment. *S. litura* fed on leaves with 2, 4 and 6 ppm of Db-Precocene I.

**Figure 3:**
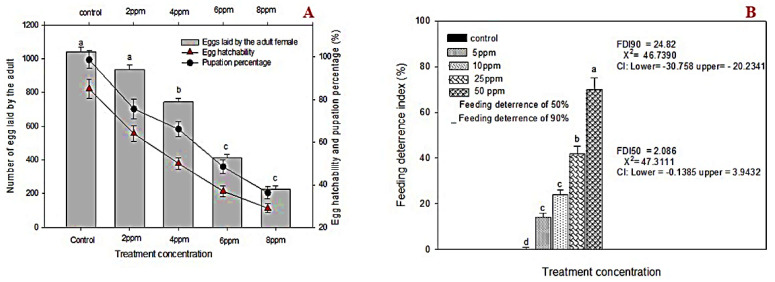
(**A**) Mean percent fecundity, egg hatch and pupation of *S. litura* after fed on leaves treated with fraction VII from *Desmostachya bipinnata*. Means (± (SE) standard error) followed by the same letters above bars indicate no significant difference (*p* ≤ 0.05) according to Tukey’s test. (**B**) Antifeedant activity of *S. litura* after treated with fraction VII from *D. bipinnata*. Means (±(SE) standard error) followed by the same letters above bars indicate no significant difference (*p* ≤ 0.05) according to Tukey’s test.

Survival was significantly decreased with increasing concentrations of Db-Precocene I (at 6 and 8 ppm). At the concentration of 6 and 8 ppm, no survival of larvae was observed on the 19th and 16th day, respectively. Over 90% mortality was observed at 20 days post treatment at 2 and 4 ppm treatments (Figure 2). The fecundity rate of female *S. litura* decreased with increasing sub-lethal concentration (2, 4, 6 and 8 ppm) of Db-Precocene I compared to control (Figure 3A). At the sub-lethal concentrations (2, 4 and 6 ppm), emergence from pupation decreased to 61% (*F*_3,16_ = 41.01, *p <* 0.019), 54% (*F*_3,16_ = 41.01, *p <* 0.020) and 41% (*F*_3,16_ = 41.01, *p <* 0.015), whereas in control, the pupal percentage was 78% (*F*_3,16_ = 41.01, *p* < 0.001). The greatest rate of reduction in pupation was at 8 ppm of Db-Precocene I, 32% (*F*_3,16_ = 41.01, *p <* 0.012).

A substantial reduction in the fecundity of *S. litura* adults occurred at 8 ppm of Db-Precocene I (228 eggs) (*F*_3,16 =_ 48.01, *p <* 0.001) compared to control (1040 eggs) (*F*_3,16_ = 48.01, *p <* 0.003). At sub-lethal concentrations, smaller reductions in fecundity occurred: at 2 ppm (935 eggs); 4 ppm (743 eggs); at 6 ppm (412 eggs) compared to control (1040 eggs).

A gradual decline in hatchability of *S. litura* egg correlated with increasing sub-lethal concentration (2–8 ppm) of Db-Precocene I. The greatest hatchability 29% occurred at the highest sub-lethal concentration compared to control 85% (*F*_3,16_ = 49.17, *p =* 0.001). Hatchability was 64%, 50% and 37% for treatments of (2, 4 and 6 ppm) of Db-Precocene I.

### 3.4. Antifeedant Activity

Feeding activity completely ceased at 50 ppm of Db-Precocene-I-treated castor leaves in fifth instar larvae of *S. litura.* The feeding activity was contrariwise to increasing concentration Db-Precocene I. The control feeding deterrence index was 0%, while the concentration of 5, 10, 25 and 50 ppm of Db-Precocene I produced percentages of feeding deterrence of 14 (*F*_4,21_ = 49.72, *p =* 0.001), 24 (*F*_4,21_ = 49.72, *p =* 0.003), 42 (*F*_4,21_ = 49.72, *p =* 0.004) and 70 (*F*_4,21_ = 49.72, *p =* 0.011), respectively (Figure 3B).

### 3.5. Earthworm Toxicity

**Figure 4:**
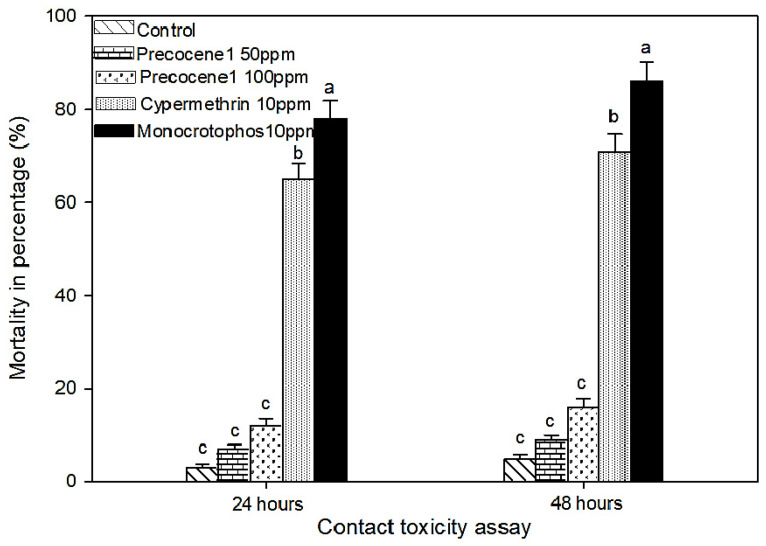
Percentage mortality of earthworm in filter paper test after treated with fraction VII and chemical pesticides. Means (± (SE) standard error) followed by the same letters above bars indicate no significant difference (*p* ≤ 0.05) according to Tukey’s test.

Regarding earthworm toxicity, there was no distinct contact toxicity observed after treatment with Db-Precocene I at 50 and 100 ppm using the filter paper test (*F*_4,21_ = 25.17, *p <* 0.001) with no significance compared to control (*F*_4,21_ = 25.17, *p <* 0.001). The percentage mortality of earthworm due to contact toxicity with Db-Precocene I at concentrations of 50 and 100 ppm after an exposure period of 24 h was 7 and 9%, and 12 and 16% after 48 h of exposure. Whereas, the chemical pesticides cypermethrin (10 ppm) and monocrotophos (10 ppm) exhibited a visible toxic effect at 24 and 48 h of exposure of 65 and 78% and 71 and 86%, respectively. Among the chemical pesticides, monocrotophos caused a more severe effect on the earthworms than cypermethrin (Figure 4).

### 3.6. Histology

**Figure 5:**
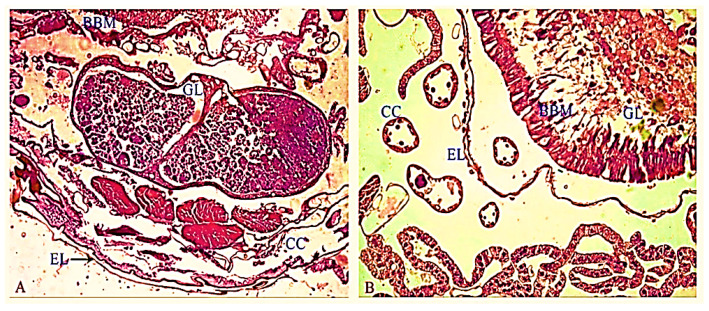
Histological section of the midgut region of fourth instar larvae of *S. litura* (**A**) control (CC—columnar cells; EL—epithelial layer; BBM—brush border membrane; GL—gut lumen). (**B**) Treated concentration of 6ppm of Db-Precocene I (CC—columnar cells; EL—epithelial layer; BBM—brush border membrane; GL—gut lumen).

The histology of midgut of *S. litura* maintained as control exhibited well distinct epithelial cells, columnar cells and conserved epithelial layer; whereas, morphological and cellular variations were observed in the midgut tissue of *S. litura* after being treated with 6 ppm of Db-Precocene I. The major changes due to Db-Precocene I included an alteration in the arrangement of the epithelial layer, enlargement and distortion of epithelial cells and formation of microvilli from the apical membrane with damage in the peritrophic membrane. Further brush border membrane and gut lumen were very much affected when compared to control (Figure 5).

### 3.7. FT-IR

**Figure 6:**
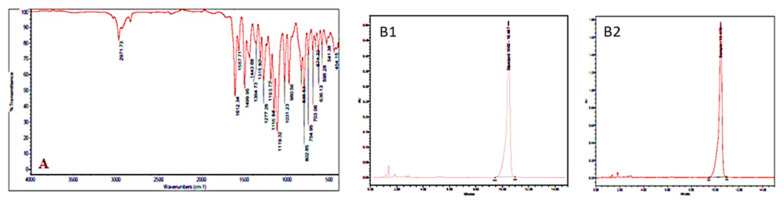
(**A**). FT-IR spectrum of fraction VII eluted from *Desmostachya bipinnata* chloroform extract 6. (**B1**). HPLC analysis of standard compound; (**B2**) HPLC analysis of fraction VII eluted from *Desmostachya bipinnata* chloroform extract.

The results showed the occurrences of various functional groups of Db-Precocene I extracted from *D. bipinnata* shown in (Figure 6A). When the extract was subjected to FT-IR, the functional group was separated, resulting in the form of peaks. The result confirmed the existence of a specific band at 2971.73 cm^−1^, showing the C-H stretching of -C-O-CH_3_, at 1364.73 cm^−1^, showing CH deformation C (CH_3_)_2_ gem dimethyl, and at 1119.32 cm^−1^, exhibiting the C-O group; also, the presence of the benzene ring was displayed by the peak’s ranges from 802.85–454.15 cm^−1^.

### 3.8. HPLC

The active fraction of *D. bipinnata* was diluted in HPLC-grade chloroform, and the sample was analyzed by injection into the HPLC. The peak obtained was comparable with standard Precocene 1 (Figure 6B). The retention time of the peak for the Db-precocene I sample (10.475) matched with the peak range of value of the Precocene I standard (10.487). The analytical result identified the compound from the chloroform extract fraction VII from *D. bipinnata* as Precocene I. To substantiate the HPLC result, a confirmation study witH-NMR technique was conducted.

### 3.9. NMR

**Figure 7:**
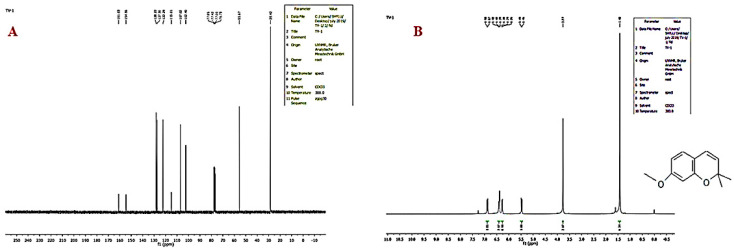
(**A**).^13^C-NMR spectrum of fraction VII (Db-Precocene I), (**B**) ^1^H-NMR spectrum of fraction VII (Db-Precocene I).

The pure compound obtained from the chloroform extract of *D. bipinnata* was subjected to ^1^H-NMR and ^13^C-NMR and spectra analyses. The values of chemical shifts were presented in ȣ scale, and the results were reported in (Figure 7A).

^1^H-NMR (CDCL_3_ MH_z_) 8 ppm: 1.43 ppm contained a singlet six proton-two methyl groups at position 2, 3.77 ppm a singlet three proton-one OCH_3_ group at position 7, 5.46 ppm a doublet (*J* = 3 Hz) one proton at position 4, and also, 6.26 ppm exhibited multiplet three protons at positions 5, 6 and 8 (Figure 7B).

## 4. Discussion

The search for eco-friendly botanical chemical alternatives has thrown a light on the unexplored wealth of plants of local origin [50,51]. The development of biopesticide products is an international concern focusing on eco-awareness and the inclusion of integrated pest management tactics [52]. As biopesticides are developed, the foundational thought should include (1) a standardized set of criteria for acceptable validation methods; (2) production outlines on how to obtain effective biopesticide products with low cost. This is indispensable to pest management for farmers that cannot rely solely on expensive synthetic pesticides, and (3) the products and production need to provide increased benefits to the farmers and community [5,53]. The untapped development of secondary plant metabolites provides a continuous rich resource to develop effective pest management products, such as biorational insecticides across many pests [53,54].

Intensive investigation with plant-derived extracts and phytochemicals have been performed for the past 30 years. In order to develop new treatments that can alleviate insecticide resistance, and to maintain pest management, the path forward depends upon standardized screening, evaluations of efficacy and safety, with the inclusion of economic benefits during production and use by farmers [55].

The agrochemicals of plants that can act as an antifeedant effectively affects the gustatory sensilla, tarsi sensilla [56] and/or odorant receptors of insects. Blocking or disruption of these host selection cues completely changes the behavior of feeding insects and results in reduced consumption, retarded growth and development and, in some circumstances, leads to death [5]. The sensilla gustatory of insects have receptors for positive and negative stimulants. The physiological mode of action on the gustatory receptors can block or prevent insects determining host acceptability information [57,58]. Many researchers are focused on the discovery and use of phytochemicals as bioactive compounds that are suitable for effective pest suppression or repellency that can be developed into insecticides [59].

For example, Yooboon et al. [60] reported on plant-based extracts with the potential for controlling *S. litura*. The ethanolic extract of *Acorus calamus* (Linn.) and *Piper retrofractum* (Linn.) were evaluated for their antifeedant and growth inhibition of *S. litura.* The study also reported the induction of behavioral changes in feeding by larval *S. litura,* resulting in longer pupation times, after feeding on leaves treated with Precocene I from *D. bipinnata.* The results were similar to those reported by Lakshmanan et al. [61].

The high mortality rate of *S.-litura*-larvae-fed Db-Precocene I leaves in our study reflect the potential to be developed into biopesticide to manage the pest *S. litura*. The rate of mortality, the reduced fecundity and egg hatch of *S. litura* fed on Db-Precocene I leaves corroborates previous reports that evaluated both Precocene I and Precocene II on *S. litura* [62,63,64,65]. While these studies report similar results, the differences observed might be the mode of activity reported for Precocene I in other studies that suggest it inhibits the ovarian DNA synthesis or interferes with vitellogenesis [66].

The structural organization of midgut of *S. litura* treated as control showed normal histological features but was drastically altered by the treatment of Db-Precocene I. Similar changes were reported when *S. litura* was treated with other types of plant toxins [44]. For example, treatment with *Swetinia mahagoni* (Linn.) leaf extracts exhibited major changes in the nature of the epithelial layer in the midgut tissue of *S. litura* [22].

In the current research, the earthworm did not appear to be affected by the Db-Precocene I treatments at the concentrations tested. The profound mortality observed from the synthetic pesticide treatments demonstrates different modes of activity, and how discovery of novel compounds may produce “environmentally friendlier” insecticides.

## 5. Conclusions

The present investigation shows that the bio-rational derivatives of *D. bipinnata* and their active metabolites produce toxic effects when fed to the tobacco cutworm, *S. litura*, at different life stages. Importantly the same concentrations were not shown to produce harmful effects on the natural soil-dwelling earthworm. For further validation of Db-Precocene I bio-activity isolated from the grass, *D. bipinnata* is needed. The activity on *S. litura* documented thus far also supports further investigation and analyses as to whether *D. bipinnata* plants have the economic potential to be a viable production source for Precocene I. If so, then there should be greater interest to move forward with development as a potential treatment to suppress this economically important pest.

## Data Availability

The data generated in the current study are available from the corresponding author on request.

## Sample Availability

Not available.
